# A new genus of soft coral (Cnidaria, Octocorallia) from the Republic of Congo (Pointe-Noire Region)

**DOI:** 10.3897/zookeys.462.8533

**Published:** 2014-12-10

**Authors:** Leen P. Van Ofwegen, Didier Aurelle, Stéphane Sartoretto

**Affiliations:** 1 Naturalis Biodiversity Center, P.O. Box 9517, NL-2300 RA Leiden, The Netherlands; 2 Aix Marseille Université, CNRS, IRD, Avignon Université, IMBE UMR 7263, 13397, Marseille, France; 3 IFREMER, Z.P. de Brégaillon, CS 20330, 83507 La Seyne-sur-mer Cedex, France

**Keywords:** Coelenterata, Cnidaria, Octocorallia, Alcyonacea, Alcyoniidea, *Alcyonium*, *Complexum*, Republic of Congo, new genus, new species

## Abstract

A new genus of soft coral from the Republic of Congo is described, *Complexum* **gen. n.** Nine West African octocoral species previously described in the genus *Alcyonium* by Tixier-Durivault (1955) are referred to this new genus, and a new species is described and figured, *Complexum pusillum* **sp. n.** The new species is characterized by having encrusting growth form and abundant spiny clubs in the surface of the polyparium. It colonizes shallow calcareous rocky banks (5 to 20 m depth) existing in coastal water of the region of Pointe-Noire. Based on molecular phylogeny this new genus is well separated from *Alcyonium* species.

## Introduction

A new species from West Africa comparable to those from the same region identified as *Alcyonium* by Tixier-Durivault (1955) was studied. Unexpectedly, in a molecular study using mitochondrial markers COI-IGR and a nuclear marker 28S we found this species to be a sister taxon of *Eunicella* instead of grouping with other *Alcyonium* species. Dr. Cathy McFadden (Harvey Mudd College, Claremont, USA) informed us she had a similar result with a species from West Africa identified as *Alcyonium monodi* Tixier-Durivault (1955). Because of these molecular data we describe a new genus to incorporate West African species previously placed in *Alcyonium*.

**Figure 1.**
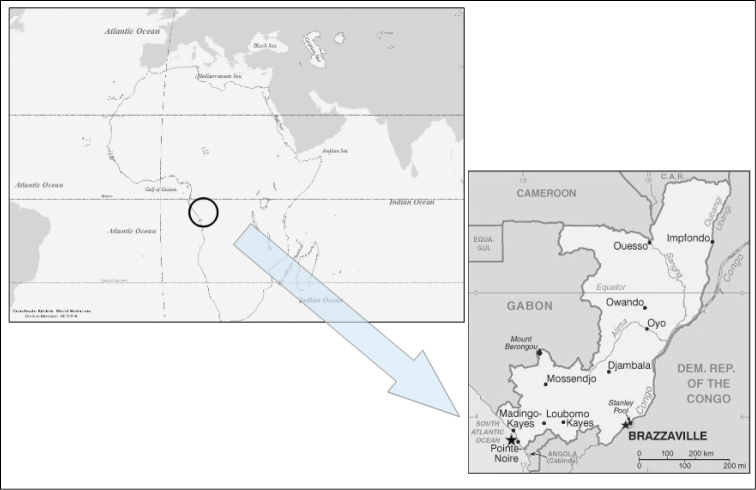
Location of sampling stations for *Complexum pusillum* sp. n. (black star).

The species here described comes from Pointe-Noire (a coastal locality of the Democratic Republic of Congo), 150 km to the north of the Congo river mouth (Fig. 1).

### Abbreviations

MNHN Muséum National d’Histoire Naturelle, Paris, France.

NBC (**RMNH**) Naturalis Biodiversity Center, formerly Rijksmuseum van natuurlijke, Historie, Darwinweg 2, P.O. Box 9517, 2300 RA Leiden, The Netherlands.

## Taxonomy

### Class ANTHOZOA Ehrenberg, 1831 Subclass OCTOCORALLIA Haeckel, 1866 Order ALCYONACEA Lamouroux, 1812 Family ALCYONIIDAE Lamouroux, 1812

#### Genus Complexum gen. n.

Taxon classificationAnimaliaAlcyonaceaAlcyoniidae

http://zoobank.org/FAE05097-0366-435B-9116-9CDB0CE30A96

##### Type species.

*Complexum pusillum* sp. n., here designated.

##### Diagnosis.

Colonies form encrusting sheets or are lobate. Polyps monomorphic and retractile. Polyps with point spindles showing an arrangement in chevrons, a kind of collaret can be present, formed by the lowest point sclerites lying horizontally. Coenenchymal sclerites are wide spindles and ovals with simple and complex tubercles. The polyparium additionally can have clubs in the surface layer, which are derived from the spindles. When preserved, colonies are white or coloured; sclerites colourless or coloured. Azooxanthellate.

##### Etymology.

From the latin *complexus*, a complex, an aggregate of parts, referring to the complex tubercles common on the coenenchymal sclerites of this genus.

##### Remarks.

The following West African species hitherto placed in *Alcyonium* are refered to the new genus: *Alcyonium caparti*, *Alcyonium globosum*, *Alcyonium gruveli*, *Alcyonium laxum*, *Alcyonium miniatum*, *Alcyonium monodi*, *Alcyonium patulum*, *Alcyonium pobeguini*, and *Alcyonium strictum*; all were described by Tixier-Durivault (1955).

#### Complexum pusillum sp. n.

Taxon classificationAnimaliaAlcyonaceaAlcyoniidae

http://zoobank.org/7CDCA56A-8F03-4525-9FCB-BC07E650E00F

**Figure 2.**
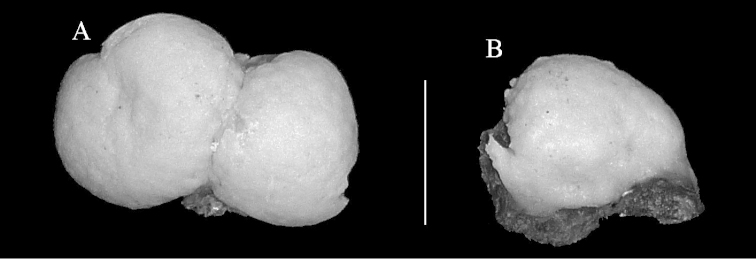
**A**, **B** *Complexum pusillum* sp. n. **A** holotype (RMNH Coel. 41604) **B** paratype (RMNH Coel. 41605). Scale bar 10 mm.

**Figure 3.**
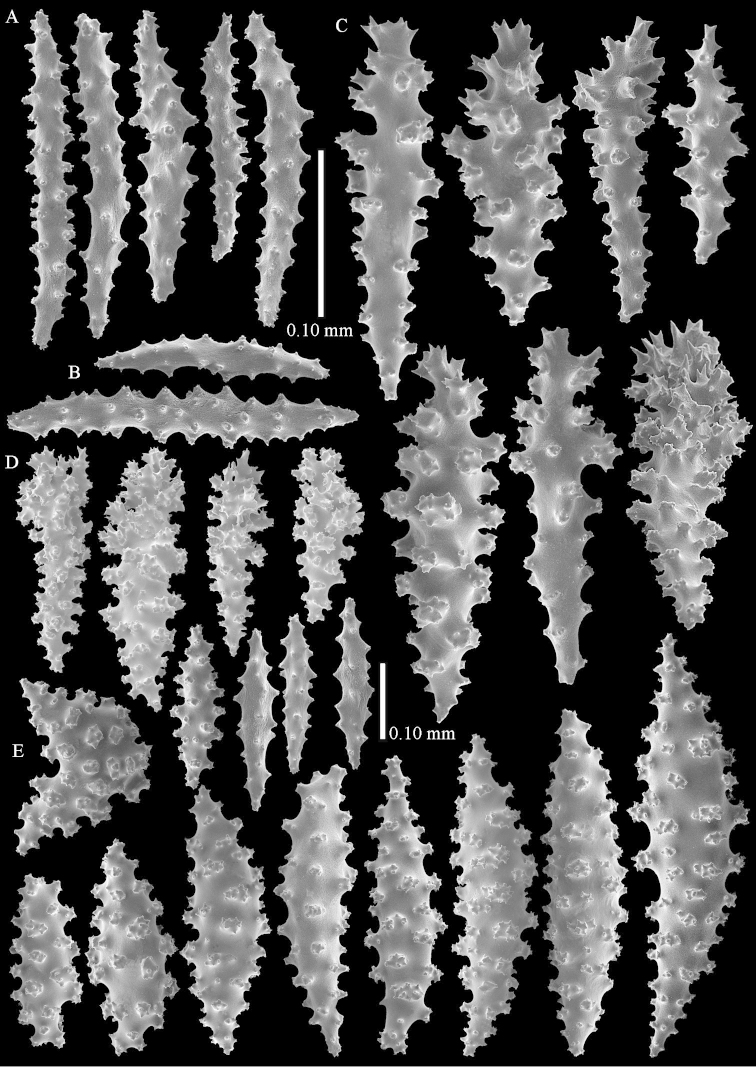
*Complexum pusillum* sp. n., holotype (RMNH Coel. 41604). **A** point spindles; **B** collaret spindles **C–D** clubs of surface layer **E** spindles of interior.

**Figure 4.**
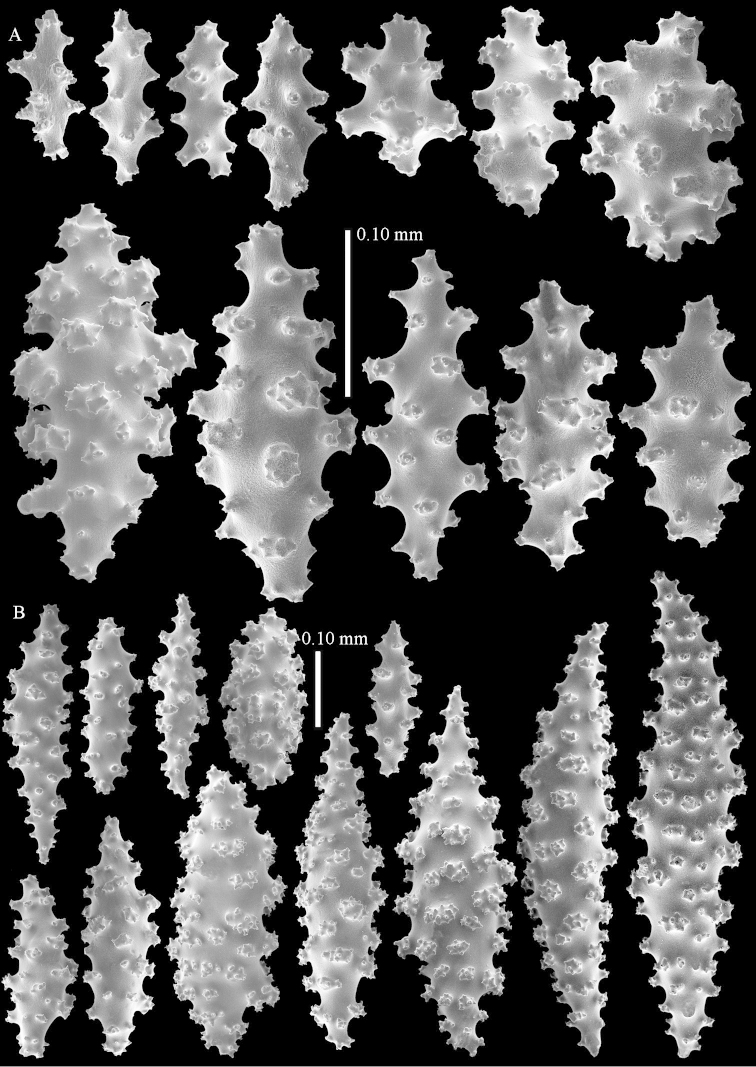
*Complexum pusillum* sp. n., holotype (RMNH Coel. 41604). **A–B** spindles of base of colony.

**Figure 5.**
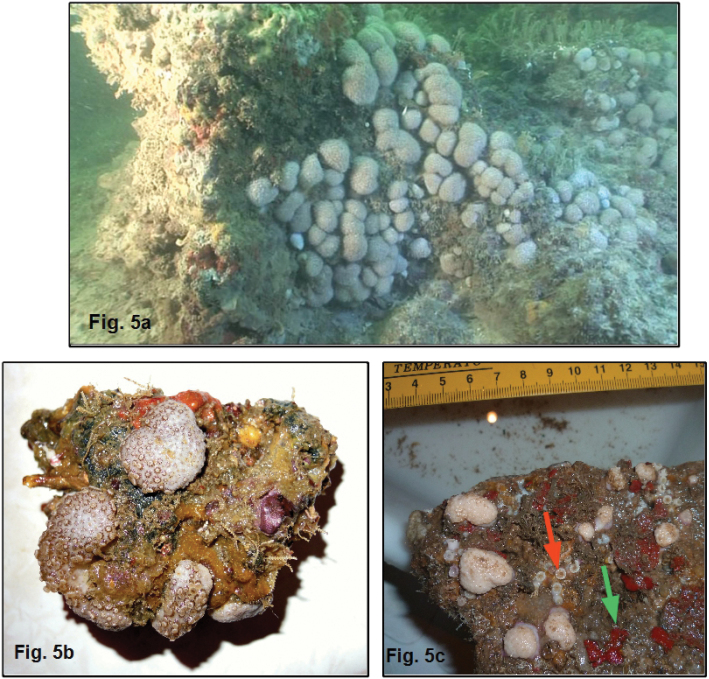
*Complexum pusillum* sp. n., Banc du Conflit, depth 10 m. **A** General view of a small patch of colonies on rocky bottom **B** Magnified view of some colonies **C** small piece of rock colonized by the new species, sponges (green arrow) and small stony corals (Polycyathus cf. senegalensis – red arrow).

**Figure 6.**
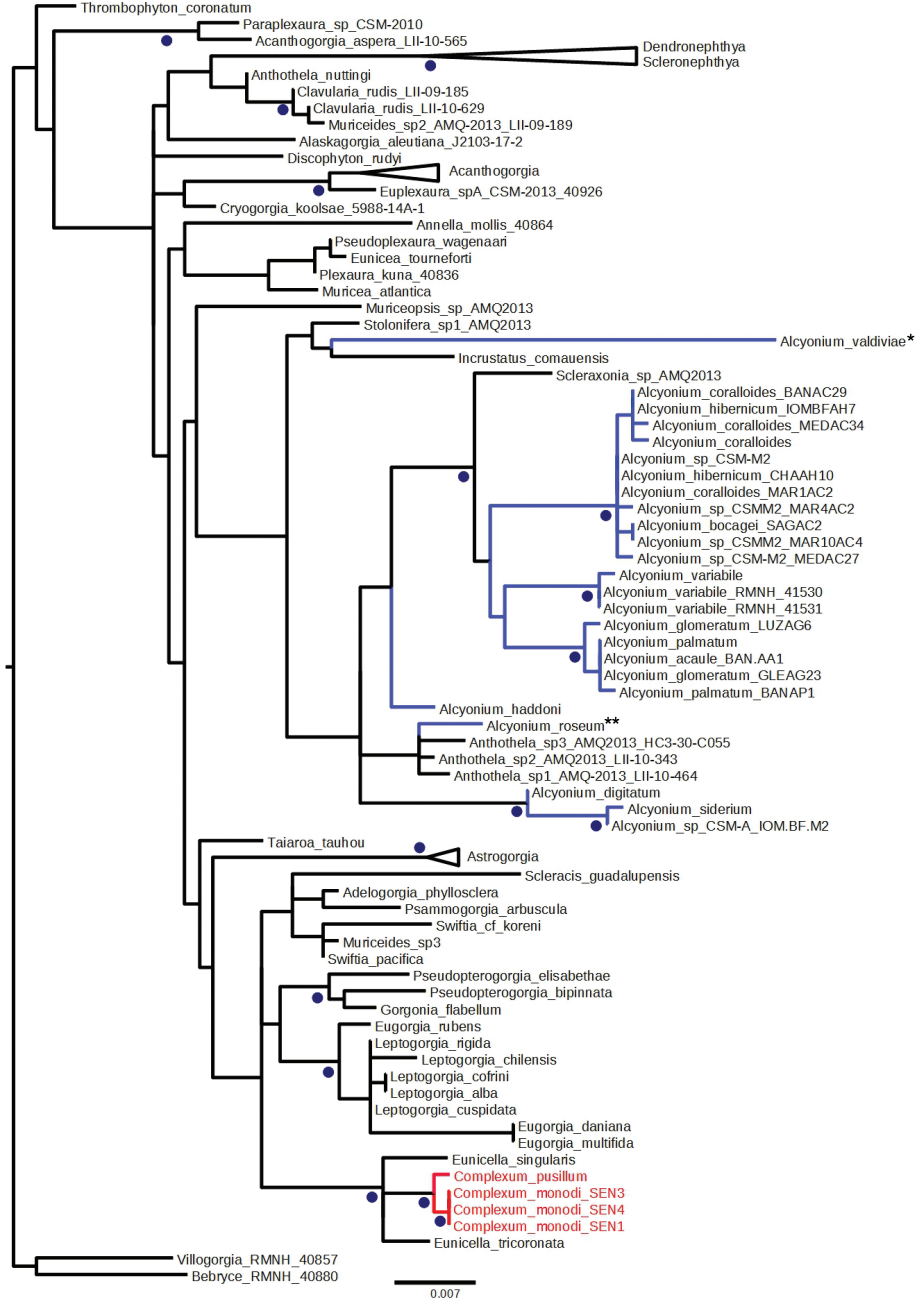
Unrooted ML phylogenetic tree based on mitochondrial COI-IGR with RaxML. Blue dots refer to groups on the right with more than 90% bootstrap support (over 1 000 bootstraps). Blue branches indicate *Alcyonium* species and red branches indicate *Complexum* species. *Alcyonium valdiviae has been transferred to the genus *Parasphaerasclera* by McFadden and Ofwegen (2013b). **Because of pre-occupation *Alcyonium roseum* has been renamed *Alcyonium varum* by McFadden and Ofwegen (2013b).

Figs 1 , 2 , 3 , 4 , 5 , 6

##### Type material.

Holotype: Congo, “Pointe Noire”, Banc Mullet, 31.I. 2013, depth -10 m, 1 colony, (RMNH Coel. 41604); paratype: same data as holotype (RMNH Coel. 41605).

##### Description.

The holotype is a colony consisting of two lobes, 2 × 1.3 cm in diameter and 1 cm thick, encrusting rock (Fig. 2A). The polyps are completely withdrawn into the coenenchyme and calyces are not present.

The anthocodiae have a collaret composed of 2–3 rows of spindles. These spindles are up to 0.15 mm long, slightly bent, and have simple tubercles (Fig. 3B). The points have spindles similar to those of the collaret, 4–5 pairs per point. They are also up to 0.15 mm long, have simple tubercles and a slightly spiny distal end (Fig. 3A). The tentacles contain no sclerites.

The surface layer of the top of the colony has clubs, up to 0.35 mm long, with complex tubercles and spiny heads (Fig. 3C–D). The interior has straight and bent spindles, up to 0.60 mm long, with simple or complex tubercles (Fig. 3E).

The base of the colony has spindles and ovals, up to 0.65 mm long, with simple or complex tubercles (Fig. 4).

##### Colour.

The preserved holotype is white, and all sclerites are colourless.

##### Etymology.

From the Latin, *pusil*, tiny, referring to the small size of the colonies.

##### Variability.

The paratype is a single lobe-like colony (Fig. 1B). The sclerites are similar to those of the holotype.

##### Habitat.

In the studied area, coastal waters show a general high turbidity due to the input of sediment and detrital and humic materials by the Congo River in the south and the Kouilou River in the north, as well as a high primary productivity in the ocean. As a consequence muddy bottoms dominate on the continental shelf (Giresse 1980). Nevertheless, shallow, cretacean, calcareous banks emerge among them, at a depth of between 5 and 20 m (Giresse and Kouyoumontzakis 1973). These rocky banks constitute large, thin slabs (no more 1.5 m high) sometimes exposed to high hydrodynamics due to the swell, tides and the occurrence of complex exchanges of water bodies up to 80 m deep (Moroschkin et al. 1970; Piton 1988). *Alcyonium pusillum* sp. n. colonizes these shallow rocky banks, mainly in cryptic positions (under overhangs and in large holes) forming small white patches (< 1 m²) easily distinguishable by SCUBA divers (Fig. 5A, B). On these hard bottoms, the associated fauna is mainly composed of gorgonians (*Eunicella*, *Leptogorgia* and *Muriceopsis* species), stony corals (Polycyathus cf. senegalensis Chevalier, 1966 in the same cryptic positions as *Alcyonium pusillum* sp. n.) and sponges (Fig. 5C).

##### Comparison with other species.

The two encrusting *Alcyonium* species previously described from Congo, *Alcyonium globosum* Tixier-Durivault, 1955, and *Alcyonium laxum* Tixier-Durivault, 1955, now referred to *Complexum*, differ from *Complexum pusillum* sp. n. in lacking clubs in the surface of the polyparium. Moreover, both these species have many lobes and coloured polyps. Other encrusting *Alcyonium* species reported from the west Atlantic now referred to *Complexum* are *Alcyonium patulum* Tixier-Durivault, 1955 and *Alcyonium strictum* Tixier-Durivault, 1955, from Mauritania. *Alcyonium patulum* resembles *Alcyonium strictum* but is red with yellow polyps and has no clubs. *Alcyonium strictum* resembles *Alcyonium pusillum* sp. n. more than any other species, it also has clubs with a spiny head, but it has many small oval sclerites in the base, and is purple with yellow polyps.

##### Molecular phylogeny.

A phylogenetic analysis has been performed, based on part of the mitochondrial COI gene and of the adjacent intergenic region (igr) which have been amplified according to McFadden et al. (2011). Additional Octocoral COI-igr sequences were retrieved from GenBank following a Blast search with the *Complexum pusillum* sequence as a query. As a comparison, *Alcyonium* spp. sequences were specifically retrieved from GenBank and included in the analysis. *Alcyonium monodi* sequences (referred to *Complexum monodi*) were kindly provided by Catherine McFadden and included in the dataset. They correspond to colonies sampled in 2012 in Senegal (10 km South of Dakar) at 15 m depth by Peter Wirtz. A phylogenetic reconstruction based on maximum likelihood (ML) has been performed with RaxML 8.1 (Stamatakis 2014) with a General Time Reversible + Gamma model and a rapid bootstrap analysis (1000 re-samplings). The nuclear gene coding for 28S ribosomal RNA has also been sequenced for *Complexum pusillum* following McFadden and Ofwegen (2013a) and the obtained sequence has been compared to other octocoral sequences thanks to a Blast search and a similar ML analysis. The COI-IGR sequence of *Complexum pusillum* has been deposited in GenBank (KP006396).

Tixier-Durivault (1955) described three other *Alcyonium* species from West Africa, *Alcyonium altum*, *Alcyonium leave* and *Alcyonium violaceum*. Verseveldt and Bayer (1988) referred *Alcyonium altum* and *Alcyonium violaceum* to the genus *Nidaliopsis* Kükenthal, 1906. It is unknown to us why Verseveldt and Bayer did not mention *Alcyonium leave*, we consider it to also belong to *Nidaliopsis*. This leaves *Alcyonium senegalense* Verseveldt & Ofwegen, 1992 from Senegal as the only *Alcyonium* species in West Africa. However, it has capstans and ovals in the coenenchyme (Verseveldt and Ofwegen 1992: figs 20–21), and therefore also does not match the current diagnosis of *Alcyonium*. We prefer to wait till molecular data of this species are available before describing another new genus to accommodate it. Superficially *Complexum* is similar to *Alcyonium*, it has the same type of colony shapes, and the sclerite arrangement in the polyps is also similar. *Alcyonium* differs in having clearly different sclerite types in surface layer and interior, radiates and club-like forms in the surface layer, and long spindles in the interior.

The phylogenetic analysis based on mitochondrial COI-IGR clearly confirmed the separation of *Complexum* from *Alcyonium* species (family Alcyoniidae) (Fig. 6). Indeed these species appear close to *Eunicella* (family Gorgoniidae; 99% bootstrap support) but the precise relationships between *Complexum* and *Eunicella* remain to be investigated with more species and the use of additional markers to get more robust information inside that group. A Blast analysis of the 28S sequence of *Complexum pusillum* in GenBank confirmed the closer relationship of this group with *Eunicella* compared to *Alcyonium* (data not shown). The current family-level taxonomy of octocorals needs extensive revision (McFadden et al. 2010) and therefore we keep the new genus in the family Alcyoniidae.

## Supplementary Material

XML Treatment for Complexum

XML Treatment for Complexum pusillum
